# Vertical ground reaction forces and loading rates during typical activities in children and adolescents: sex- and maturity-specific considerations for bone health

**DOI:** 10.3389/fendo.2026.1748455

**Published:** 2026-03-10

**Authors:** Gemma Brailey, Brad Metcalf, Lisa Price, Victoria Stiles

**Affiliations:** University of Exeter, Department of Public Health and Sport Sciences, Exeter, United Kingdom

**Keywords:** adolescents, bone health, children, force loading rate, ground reaction force

## Abstract

**Introduction:**

Despite physical activity (PA) during childhood being one of the most important modifiable factors influencing osteoporosis risk, the ground reaction forces (GRFs) associated with typical physical activities remain largely unknown, and the influence of sex and maturity on these factors is rarely considered. This study aimed to quantify the GRF and force loading rates during typical everyday activities in children and adolescents.

**Methods:**

A total of 282 children (127 boys, 142 girls; aged 8–16 years) completed walking, running, jumping and hopping on a portable force plate. Maturity was determined using maturity offset relative to peak height velocity (PHV) and categorised as Pre- or Post-PHV. Peak vertical force (PVF) and average loading rate (ALR) were extracted from force-time histories. Linear mixed-effects regression models were fitted separately for PVF and ALR, including fixed effects for activity, sex, maturity group, their two- and three-way interaction terms, and force-plate type, with participant (ID) as a random effect.

**Results:**

There was a significant three-way interaction for both PVF (F_(4,225)_= 4.15, *p* = 0.003) and ALR (F_(4,225)_= 5.54, *p* < 0.001), indicating that the effect of maturity differed across activities and between sexes. For locomotor tasks, PVF decreased significantly from Pre- to Post-PHV in both sexes during walking (both *p*’s <0.001) and in boys during running (*p* < 0.001), with a similar, but non-significant trend in girls (*p* = 0.291). The ALR also decreased during running in both sexes, reaching significance in boys (*p* < 0.001) and borderline significance in girls (*p* = 0.057). The jumping activities showed divergent sex-specific patterns. Boys tended to maintain or increase PVF and ALR with maturation in the low and high jump activities (*p* = 0.004 –0.914), whereas girls showed consistent reductions from Pre- to Post-PHV (*p* < 0.001-0.057).

**Discussion:**

Findings demonstrate that children and adolescents do not always experience the same osteogenic stimulus for a given activity. Consideration of sex- and maturity-specific loading profiles may therefore be important for designing and interpreting bone-benefitting interventions that deliver an appropriate osteogenic stimulus across childhood and adolescence.

## 1 Introduction

The mechanical environment of the growing skeleton is a key determinant of lifelong bone strength (1). Weight bearing physical activity (PA) is one of the most important modifiable determinants of bone health and lifelong osteoporosis risk (1). Despite being considered a condition of old age, the antecedents of osteoporosis begin in childhood, with around a third of adult bone mass being accrued during the two years surrounding the adolescent growth spurt (2). Optimising mechanical loading during this critical window can therefore help to maximise peak bone mass and delay or prevent osteoporosis later in life (3). Whilst a large body of evidence supports the beneficial and prophylactic effects of PA on skeletal development in children and adolescents (1, 3–5), few studies have directly quantified the mechanical forces generated by everyday activities. Without this, it remains difficult to identify the specific loading features that are most osteogenic at a given age or to translate them into evidence-based activity thresholds for bone health promotion in this population (6). In adults, mechanical loading thresholds have been proposed to classify impact intensity - for example, activities producing >4 body weights (BW) are typically considered ‘high-impact’ (7, 8), yet comparable thresholds for youth have not been established.

A small number of intervention studies that enhanced bone health in youth populations have quantified the ground reaction forces (GRFs) associated with various high-impact jumping activities (thought to be most osteogenic), including drop jumps, countermovement jumps, plyometric jumps, and jumping jacks (9–13). However, substantial variation in reported GRFs for ostensibly similar activities have been observed. For example, Fuchs et al. (10) reported peak vertical GRFs of 8.8 body weights (BW) for a drop jump from a 61 cm box in 6–10-year-old children, whereas McKay et al. (12) found forces almost half this magnitude (4.7 BW) for a drop jump from a comparable height (50 cm) in 8–11-year-olds. Whilst some of this variation may reflect differences in methodology, sample characteristics, or the inherently variable jumping performance of children (14), the wide range in reported forces makes it difficult to define osteogenic loading magnitudes with confidence. The same pattern is evident in the limited studies that have assessed force loading rates (9, 12). Loading rate is an important, yet often overlooked proxy measure of strain rate, and alongside load magnitude, is a key determinant of bone adaptation (15). Variability in reported loading rates for similar activities further complicates efforts to determine osteogenic activities in youth.

Although intervention studies have largely focused on high-impact jumping activities that are believed to be most osteogenic, lower-level impacts may also be osteogenic if performed with sufficient frequency (15, 16). Several descriptive studies have therefore sought to quantify the GRFs associated with a range of everyday activities in children and adolescents, providing valuable insight into the magnitude of mechanical loading during walking, running, jumping and hopping (17–21). However, these studies are limited by small sample sizes (between 8–50 participants) that are composed primarily of pre- and early-pubertal children, and most do not account for the influence of sex or biological maturity on loading patterns. As children mature, changes in bone geometry, muscle strength and tendon stiffness, together with broader biomechanical and neuromuscular adaptations, enhance lower limb function and influence movement patterns and the way forces are generated and absorbed during movement (22, 23). These developmental changes occur differently in boys and girls, leading to sex-specific variations in lower extremity kinematics and kinetics (24). Ignoring these influences risks misinterpreting the osteogenic potential of mechanical loading in youth (6).

To advance understanding of how weight-bearing PA influences bone health across growth and maturation, it is essential to quantify how both sex and biological maturity affect key loading characteristics. Accounting for these factors will improve the interpretation of mechanical loading so that the osteogenic potential of loading experienced from an activity can be better estimated in youth. This study therefore aimed to quantify peak impact forces and rates of loading during walking, running, jumping and hopping activities in a large sample of children and adolescents, and to determine whether these measures differ according to sex and maturity. A more nuanced understanding of the loading characteristics of everyday activities in children and adolescents will provide critical context for interpreting how weight-bearing PA contributes to skeletal development, informing both mechanistic models of bone adaptation and the design of targeted interventions and public health strategies aimed at optimising bone health during this critical window of opportunity.

## 2 Methods

A total of 282 children and adolescents aged 8–16 years were recruited from four primary and three secondary schools in and around Exeter, Devon (UK). As whole classes were approached within the participating schools, the exact number of children invited to attend was not recorded, and the final sample comprises those who returned consent forms and completed testing. Children were sent home with a study information pack that contained all key study documentation (information sheet, parental consent form, child assent form, medical screening questionnaire) and were given a period of two weeks in which they could return the forms and confirm participation in the study. Prior to data collection, consent was obtained from a parent/guardian and written assent was obtained from children, with children providing reconfirmation of assent on the day of data collection. Ethical approval for the study was granted the University of Exeter Sport and Health Sciences Ethics Committee (ref: 170315/B/01 and 171206/B/10).

Data collection was conducted in two waves, with primary school children (aged 9-11) completing the study in June and July of 2017 (Wave 1) and secondary school children (aged 11-16) completing the study from March to July of 2018 (Wave 2). To minimise disruption, data collection was scheduled around timetabled physical education lessons and took place in the main school hall/sports hall (un-sprung floor). Anthropometric measures (body mass, stature and sitting height) were taken following standardised procedures (25). Stature and sitting height were measured to the nearest 0.1cm using a portable stadiometer (Leicester Height Measure; Seca, Birmingham, UK), and body mass was measured to the nearest 0.1 *kg* using an electronic scale (Seca 7802317004, Birmingham, UK). Two readings were obtained for each measure and averaged; if values differed by more than 0.4 cm or 0.4 kg, a third was taken and the median reported. Body mass index (BMI) was calculated as body mass (kg) divided by height (m) squared (kg/m²), and leg length as standing height minus sitting height. Biological maturity was estimated as the number of years each participant was from their predicted age at peak height velocity (PHV), calculated using the sex-specific equations developed by Mirwald and colleagues (26). This value (termed maturity offset) was derived by subtracting the predicted age at PHV from the child’s chronological age at data collection.

Following a brief warm-up, demonstration of activities by a member of the research team and familiarization with each activity, participants performed 5 standardised activities (walking, running, low jumps, high jumps and single-leg hopping) on a portable force platform in a randomised order. Prior to data collection, a member of the research team demonstrated each activity whilst listening to the corresponding metronome beat. Participants were instructed to perform each activity in time with the researcher, with emphasis placed on consistency of technique and timing rather than maximal or competitive effort. Walking was described as a “comfortable, brisk walking pace”, while running was described as a “fast jog” that was “not an all-out maximal sprint”. Low jumps were described as small two-footed “bunny hops - the smallest jump you can imagine with both feet off the floor”, while higher jumps were described as “something between the smallest jump you can imagine and the highest jump you can do”. Single leg hopping was performed on the preferred leg using a similar small amplitude hopping motion. Each participant practiced all activities alongside the researcher prior to data collection and received feedback to ensure close adherence to the metronome timing and correct technique.

Wave 1 data was collected using an AccuSway PLUS platform (Advanced Mechanical Technology Inc., Watertown, MA, USA; 50.2 × 50.2 × 4.5 cm), sampling at 200 Hz using AMTI NetForce software (version 3.5.3). In Wave 2, an AccuPower platform (Advanced Mechanical Technology Inc.; 102 × 76.2 × 12.5 cm) was used, sampling at 1000 Hz using AccuPower software (version 2.0). Each force platform was installed flush at the midway point of a 10 m runway that was topped with a 10mm depth of EVA foam. Children wore sports shoes and performed shuttles of walking and running on this runway at fixed cadences (a metronome was set to 120 beats per minute for walking and 190 beats per minute for running) for 60 s. All walking and running trials were performed to a metronome-set cadence to reduce variability in movement timing, which may influence GRFs and loading rates. Self-selected cadence was not assessed as the study aimed to prioritise controlled comparisons across sex and maturity groups.To ensure that children made correct contact with the plate without altering their natural gait, a member of the research team performed the activities alongside them and was able to adjust their distance from the centre to ensure correct contact was made. If a participant did not make correct contact with the force plate during walking or running, the researcher subtly adjusted the starting distance on the runway and repeated the trial, ensuring correct contact without altering the child’s gait pattern.

The jumping (low jumps: approximately 2–5 cm; 120 beats per minute; and higher jumps: >5 cm; 90 beats per minute) and single-leg hopping (2–5 cm, 130 beats per minute) activities were completed continuously on the force plate for 10 s. A member of the research team zeroed the force plate prior to the start of each activity and before a participant stepped onto the plate to collect jumping data on the spot. Participants were asked to maintain an upright posture, use a slight knee bend for take-off and landing, and move arms naturally. If jumping or hopping movements were markedly out of sync with the metronome or demonstrated inconsistent technique, the researcher paused the trial, provided verbal feedback and restarted the activity. Participants were bought to the testing area in groups of three or fewer to minimise disruption. Activities were not performed directly in front of peers, as other group members were engaged in anthropometric measurements or assent procedures during testing.

Bone adaptation is influenced by both the magnitude and rate of mechanical strain resulting from gravitational and muscular forces acting on the skeleton (27). Vertical ground reaction force provides a practical surrogate for strain magnitude, as it reflects the external load applied to the skeleton. The rate at which these forces are applied (represented by the loading rate) is also an important determinant of adaptation in bone (28, 29). All force variables reported in this study were derived from the landing phase of each activity. Accordingly, the outcomes derived from the force data were the peak vertical ground reaction force (PVF), expressed relative to body weight (BW) calculated by the equation


$$\frac{\text{output force }(\text{N})}{\text{mass }(\text{kg})\text{ x }9.81\text{ m}.{\text{s}}^{-2}}$$

and the average loading rate (ALR; BW/s) calculated by the equation


$$\frac{\text{peak vertical force }(\text{BW})}{\text{time of peak force }(\text{s})-\text{start time of ground contact }(\text{s})}$$

Each individual step during the walking and running trials was visually examined in Excel to verify that clear and complete contact had been made with the force plate. Trials in which the participant only partially contacted the plate were discarded. The period of ground contact was defined as the duration for which vertical force exceeded 10 N, beginning when the trace rose above this value to the point it fell below again. A minimum of four valid steps were retained for both the walking and running activities. For the jumping and hopping activities, the full 10-second force-time histories were reviewed to identify distinct take-off and landing phases. Ground reaction force variables were then computed as mean values across roughly eight consecutive jumps or hops that demonstrated consistent technique and complete contact with the plate.

All analyses were conducted in R (version 4.5.1). Descriptive statistics (mean ± SD) were calculated for age, anthropometric characteristics, and maturity offset for the whole sample and for boys and girls separately. Maturity offset was dichotomised into Pre-PHV (all those with a negative maturity offset value) and Post-PHV (all those with a positive maturity offset value) as described by Mirwald et al. (26) to represent distinct stages in biological maturation. Histograms of the PVF and ALR data were inspected prior to analysis and revealed that ALR was very positively skewed (skewness = 2.22). This variable was therefore logarithmically transformed for the analysis.

Linear mixed-effects regression models were fitted separately for PVF and ALR using the *lmer* function in the *lme4* package for R. Fixed effects were activity (walking, running, low jumps, high jumps, single-leg hopping), sex (male, female), maturity group (Pre- or Post-PHV), their two- and three-way interaction terms, and force-plate type to adjust for any potential differences between the data collection waves that used different models of force plate and sampling rates. ID (participant) was included as a random effect to account for repeated measures. Estimated marginal means (EMMs) were calculated for each of the 20 sex x maturity group x activity combinations, adjusted for force plate type. Model coefficients indicated whether there were differences between the pre- and post-PHV groups within each sex by activity combination. As ALR was analysed on the natural log scale, model coefficients were back-transformed by exponentiation to express effects as ratios of geometric means, representing the relative (percentage) difference between Post- and Pre-PHV groups.

Model assumptions were checked through inspection of residual diagnostic plots. Residuals were approximately normally distributed (QQ plots), though the residual vs fitted plot demonstrated that there was some heteroscedasticity. To account for this, analyses were repeated using cluster-robust (CR2) standard errors (SEs), clustering by participant to provide robust standard errors that allow for differences in residual variance across participants. This approach does not alter model estimates but provides corrected standard errors, test statistics, and *p* values. The robust results were very similar to the original models, indicating that the overall pattern of results and their interpretation remained unchanged. All statistics were therefore obtained from the linear mixed-effects regression with robust SEs model. Statistical significance was set at *p* < 0.05. Data visualisations were produced using *ggplot2*, showing model-adjusted means with 95% confidence intervals to illustrate the interaction effects.

## 3 Results

### 3.1 Sample characteristics

Descriptive statistics for the whole sample, and for boys and girls separately are summarised in **Table 1**. The final sample consisted of 269 participants (127 boys; 142 girls). Based on maturity offset, 143 participants were classified as Pre-PHV (87 boys, 56 girls) and 126 as Post-PHV (40 boys, 86 girls). A technical error with the force plate in the first wave of data collection resulted in the loss of GRF data for 13 participants. The final study sample had a mean age of 12.3 (± 2.0) years, and boys and girls were similar in terms of age, height, leg length and mass. As expected, girls had a significantly lower predicted age of PHV compared to boys (12.02 vs. 13.60 years, *p* < 0.05).

**Table 1:** Descriptive characteristics for the whole study sample, and for boys and girls separately (mean (SD)).

Participant Characteristic	All (n= 269)	Male (n=127)	Female (n=142)
Age (yr)	12.3 (2.0)	12.3 (2.1)	12.3 (1.9)
Height (m)	1.54 (0.14)	1.55 (0.15)	1.54 (0.12)
Leg length (m)	0.74 (0.07)	0.75 (0.08)	0.73 (0.07)
Weight (kg)	46.01 (13.50)	45.15 (13.75)	46.79 (13.27)
BMI (kg/m^2^)	18.97 (3.12)	18.43 (2.87)	19.45 (3.27)
Predicted age at PHV (yr)	12.8 (1.0)	13.6 (0.7)	12.0 (0.6)
Maturity offset (yr)	-0.44 (1.93)	-1.28 (1.86)	0.31 (1.68)

### 3.2 Peak vertical force

The model including the three-way Activity × Sex × Maturity interaction term provided a significantly better fit than the model containing only two-way terms (χ²_(4)_ = 35.9, *p* <.001). A Type III test confirmed that the three-way interaction was significant (F_(4, 225)_ = 4.15, *p* = 0.003), demonstrating that the effect of maturity on PVF differed across activities and between sexes.

**Figure 1:**
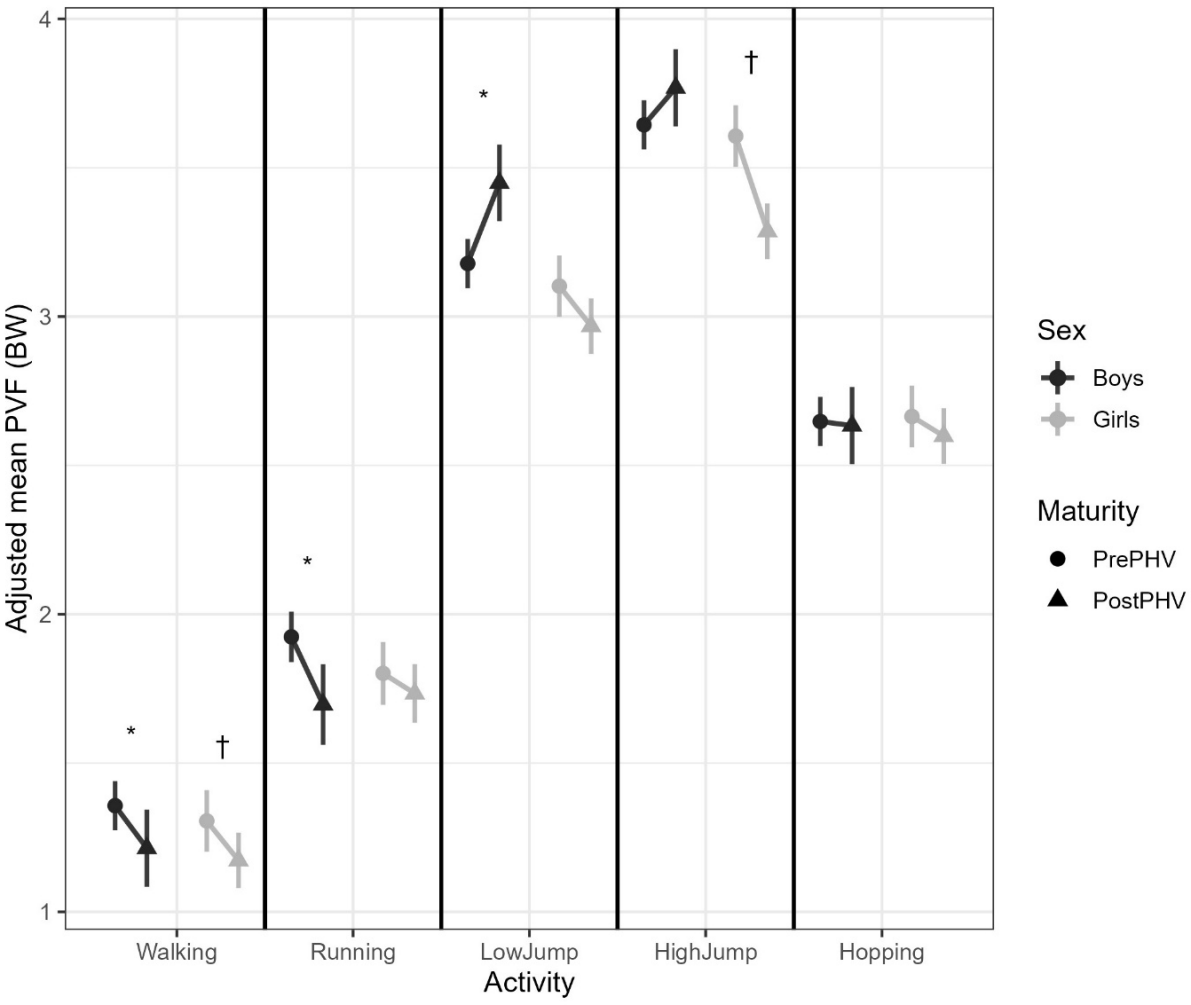
Adjusted mean peak vertical force (PVF; Body Weights) during walking, running, low jumps, high jumps and hopping, shown separately for boys and girls before (Pre-PHV) and after peak height velocity (Post-PHV). Each line connects model-adjusted estimated marginal means (EMMs), with error bars representing 95% confidence intervals from mixed-effects models adjusted for force-plate type and clustered by participant. * indicates a significant difference between Pre- and Post-PHV in boys (p <.05). † indicates a significant difference between Pre- and Post-PHV in girls (p <.05). PVF, Peak Vertical Force; PHV, Peak Height Velocity; BW, Body Weights.

Estimated marginal means (EMMs) and their pairwise contrasts were used to explore the significant three-way interaction between activity, sex, and maturity (**Table 2**; **Figure 1**). Simple effects of maturity (Pre- vs Post-PHV) were examined within each sex × activity combination. There was a significant decrease in PVF from Pre- to Post-PHV in both sexes for walking (both *p*’s < 0.001) and in boys only for running (boys *p* < 0.001; girls *p* = 0.29). The trend in PVF from Pre- to Post-PHV for each sex was more variable for the jumping activities. For example, boys significantly increased their PVF from Pre- to Post-PHV in the low jumping activity (*p* = 0.004) but showed no clear change in the higher jumping activity (*p* = 0.31). In contrast, girls showed a reduction in PVF from Pre- to Post-PHV in the higher jumps (*p* = 0.003) and a trend for a reduction that approached significance in the low jump activity (*p* = 0.057). No significant maturity effects were observed amongst sexes for hopping (boys *p* = 0.81, girls *p* = 0.25 for girls, respectively).

**Table 2:** Estimated maturity-related differences in peak vertical force and average loading rate for each activity and sex from the linear mixed-effects model, adjusted for force plate type and clustered by participant.

Activity	Sex	PVF	95% CI (Post – Pre PHV)	*p*	ALR (Post - Pre PHV)	95% CI (Post – Pre PHV)	*p*
		(Post - Pre PHV)					
Walking	Boys	**-0.143**	-0.209, -0.076	<0.001*	-0.012	-0.088, 0.063	0.747
	Girls	**-0.132**	-0.201, -0.064	<0.001*	-0.043	-0.127, 0.041	0.315
Running	Boys	**-0.227**	-0.343, -0.112	<0.001*	**-0.260**	-0.392, -0.128	<0.001*
	Girls	-0.068	-0.193, 0.058	0.291	-0.131	-0.266, 0.004	0.057
Low jumps	Boys	**0.271**	0.087, 0.454	0.004^†^	0.099	-0.024, 0.221	0.114
	Girls	-0.134	-0.273, 0.004	0.057	**-0.121**	-0.224, -0.018	0.021*
Higher jumps	Boys	0.124	-0.118, 0.367	0.314	0.007	-0.123, 0.138	0.914
	Girls	**-0.320**	-0.528, -0.112	0.003*	**-0.281**	-0.398, -0.165	<0.001*
Hopping	Boys	-0.014	-0.128, 0.100	0.810	**-0.135**	-0.234, -0.037	0.007*
	Girls	-0.066	-0.177, 0.046	0.250	**-0.232**	-0.364, -0.099	<0.001*

*= significant decrease from Pre- to Post-PHV; ^†^ = significant increase from Pre- to Post-PHV.

Pre-PHV boys n= 87; Post-PHV boys n= 40; Pre-PHV girls n= 56; Post-PHV girls n=86.

PHV = peak height velocity; SE = standard error estimated using cluster-robust methods; PVF= Peak vertical force, expressed in BodyWeights (BW), where 1 BW is equivalent to the participant’s body mass x 9.81 m.s^-2^; ALR, Average loading rate, expressed in BW/s on the log-transformed scale (negative values correspond to lower geometric mean loading rates Post-PHV).

Significant differences are highlighted in bold. All CIs and *p* values are from cluster-robust outputs.

Negative differences indicate lower force or loading Post-PHV (i.e., a decline from Pre- to Post-PHV), whereas positive values indicate higher force or loading Post-PHV.

Model-adjusted mean PVF values (EMMs; derived from the mixed-effects model, adjusted for force-plate type and clustered by participant) are presented in **Table 3** and illustrate the loading magnitudes that underly the observed maturity effects. These estimates offer a practical reference for the magnitude of vertical loading experienced during everyday movement tasks in youth before and after PHV. Adjusted mean PVF values from the mixed-effects model (EMMs; **Table 3**) ranged from 1.17–1.36 BW during walking to 3.29–3.77 BW during the high jump activities, demonstrating that the mechanical loading incurred increased progressively from ambulatory to jumping activities. Boys tended to produce slightly higher PVF than girls at both Pre- and Post-PHV. Maturity-related changes were small for walking and running (~ 0.1–0.2 BW) but were larger for jumping activities. For example, boys PVF increased in the low jumps by approximately 0.3 BW, whereas girls PVF decreased by around 0.3 BW in the high jumping activity. These model-adjusted estimates (**Figure 1**; **Table 3**), highlight that whilst PVF increased with activity intensity, the direction of maturity-related changes differed by sex and activity.

**Table 3:** Model-adjusted (estimated marginal) means for peak vertical force and average loading rate during walking, running, and jumping activities for males and females before and after peak height velocity.

Activity	Sex	PVF	Post-PHV mean	Δ (Post – Pre)	ALR	Post-PHV mean	Δ (Post – Pre)
		Pre-PHV mean			Pre-PHV mean		
Walking	Boys	1.36	1.21	−0.15	11.35	11.21	-0.14
	Girls	1.31	1.17	−0.14	11.20	10.73	-0.47
Running	Boys	1.92	1.70	−0.22	85.90	66.24	-19.65
	Girls	1.80	1.73	−0.07	85.75	75.20	-10.55
Low jumps	Boys	3.18	3.45	0.27	23.05	25.44	2.39
	Girls	3.10	2.97	−0.13	22.53	19.96	-2.57
Higher jumps	Boys	3.64	3.77	0.13	30.54	30.76	0.22
	Girls	3.61	3.29	−0.32	32.52	24.55	-7.97
Hopping	Boys	2.65	2.63	−0.02	22.45	19.61	-2.84
	Girls	2.66	2.60	−0.06	26.65	21.14	-5.51

**Figure 2:**
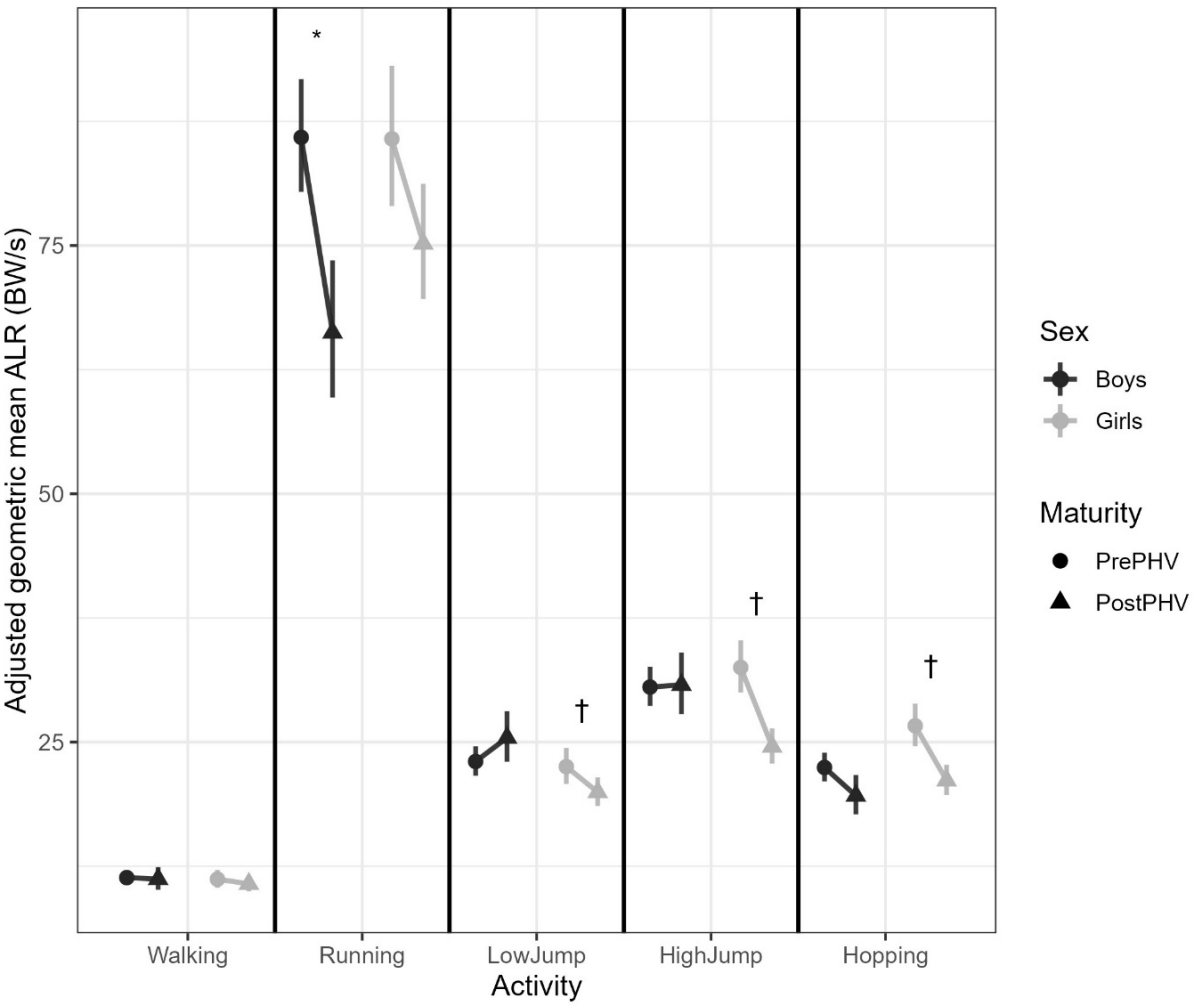
Adjusted geometric mean average loading rate (ALR; BW·s^−1^) during walking, running, low jumps, high jumps, and hopping, shown separately for boys and girls before (Pre-PHV) and after peak height velocity (Post-PHV). Points and error bars represent back-transformed EMMs and 95% confidence intervals from mixed-effects models adjusted for force-plate type and clustered by participant. * indicates a significant difference between Pre- and Post-PHV in boys (p <.05). † indicates a significant difference between Pre- and Post-PHV in girls (p <.05). ALR, Average Loading Rate; PHV, Peak Height Velocity; BW, Body Weights.

Means correspond to the adjusted values shown in **Figure 1** and **2**.

PHV, peak height velocity; PVF, Peak vertical force, expressed in BodyWeights (BW), where 1 BW is equivalent to the participant’s body mass x 9.81 m.s^-2^; ALR, Average loading rate (model-adjusted geometric means from the log-ALR model (back transformed)), expressed in BW/s.

Pre-PHV boys n= 87; Post-PHV boys n= 40; Pre-PHV girls n= 56; Post-PHV girls n=86

Values are derived from mixed-effects models, adjusted for force-plate type and clustered by participant. For the changes in loading magnitude and rate across maturity, negative values indicate lower loading post-PHV and positive values indicate higher loading post-PHV. These adjusted means provide descriptive context for the interaction patterns illustrated in **Figure 1**. and **Figure 2**. and the statistical contrasts reported in **Table 2**.

### 3.3 Average loading rate

The three-way Activity x Sex x Maturity interaction term resulted in a significantly better model fit (χ²_(4)_ = 21.02, *p* = 0.0003) and had a significant three-way interaction term (F_(4, 225)_ = 5.54, p = 0.0002). Consistent with the PVF findings, the effect of maturity on ALR therefore also differed across activities and between sexes.

The simple effects of maturity within each sex x activity combination (**Table 2**; **Figure 2**.) demonstrated that there was no maturity effect for walking in either sex (boys *p* = 0.75; girls *p* = 0.31), but ALR decreased significantly from Pre- to Post-PHV in running for boys (*p* < 0.001) and showed a smaller, borderline non-significant reduction in girls (*p* = 0.057). The jumping activities also showed a less consistent pattern between sexes. For example, ALR decreased from Pre- to Post-PHV in girls for both the low and high jump activities (*p* = 0.021 and <0.0001, respectively), whereas for boys, low jumps had a non-significant upward trend in ALR from Pre- to Post-PHV (*p* = 0.114) and no clear change in the high jump activity (*p* = 0.91). For hopping, both boys and girls showed a reduction in ALR from Pre- to Post-PHV, however, this was only significant in girls (*p* = 0.006).

The model-adjusted mean ALR values (EMMs; back transformed; **Table 3**.) demonstrate that ALR values were approximately 11 BW·s^−1^ during walking. Running had the highest loading rates, reaching ~86 BW/s in Pre-PHV boys and girls and then reducing to 66 BW/s in boys and 75 BW/s in girls, respectively Post-PHV. Of the jumping activities, high jumps had the highest ALR of ~30 BW/s in both Pre- and Post-PHV boys and reduced from 32 to 24 BW/s from Pre- to Post-PHV in girls. There was a trend for boys to have slightly higher ALR than girls and this was particularly evident at Post-PHV for the most impactful high jump activity, where an ALR of 30.76 BW/s was reported for boys, compared to 24.55 BW/s in girls. These model adjusted estimates demonstrate that whilst absolute loading rates increase with the activity intensity, the direction of maturity-related changes varies by sex and the activity performed.

## 4 Discussion

Quantifying the force-loading characteristics of everyday activities is a crucial yet often overlooked step in understanding the characteristics of PA that are beneficial to bone health in children and adolescents. This study sought to quantify the ground reaction force and force loading rates during walking, running, jumping and hopping in a large sample of children and adolescents, and determine whether these differed due to sex and/or maturity. Findings demonstrated that the direction and extent of maturity-related changes differed between sexes and across activities. Loading tended to decrease from pre- to post-PHV during locomotor tasks, but patterns were less consistent for jumping activities. Boys maintained or increased loading with maturation during jumping, whereas girls exhibited reductions in both peak force and loading rate, especially at higher jump intensities. These findings highlight the importance of accounting for sex and maturity when characterising mechanical loading in youth and determining osteogenic thresholds, as developmental differences can influence the loads experienced during everyday movements.

The GRFs and loading rates reported in this study are comparable to those reported previously for walking (20, 21, 30), running (20, 21), and similar jumping and hopping activities (12, 19, 31). However, this study goes a step further by providing a more nuanced understanding of how GRFs and loading rates may vary during growth and maturation, which has rarely been considered in previous work. For the ambulatory walking and running activities, PVF tended to decrease from Pre- to Post-PHV in both boys and girls, whereas ALR remained constant for walking but decreased from Pre- to Post-PHV in both sexes for running. There is a paucity of evidence investigating the influence of sex and maturity on force production and spatiotemporal parameters during walking and running in this population (23). Beck et al. (30) investigated walking patterns in 51 children aged between 11 months and 15 years and reported that GRF decreased significantly between children in the youngest age group (1–3 years) and those aged 4–5 years but remained consistent with no significant differences between the ages of 5–15 years. Similar findings were also reported by Diop et al. (32), who found no significant differences in PVF during walking after the age of 8 years. A similar pattern was also observed for running, where there was a lack of significant differences in PVF between children aged 7-8, 9–10 and 11–12 years of age when running at 2.2 m/s (33). However, it is of importance to note that the aforementioned studies have compared children at the same chronological, rather than biological age, and (with the exception of (33), have included small sample sizes that may lack statistical power to detect such differences. When studying gait in children and adolescents, it is more appropriate to make comparisons based on biological age, as most intrinsic factors that influence gait are in fact dependent on biological age (23). With advancing biological age, children experience an improvement in lower-limb performance (22) and experience greater pre-activation and engagement of the stretch reflex, which serves to effectively reduce stiffness during landing and the peaks in GRF during ground contact (34, 35). Based on this, it is therefore unsurprising that the present study found a reduction in both PVF and ALR in more mature children.

Findings for the jumping activities were less consistent. Girls’ force loading characteristics followed the same pattern as running, with a reduction in both force and loading rate from Pre- to Post-PHV for low and high jumping activities, whereas boys showed an increase in PVF from Pre- to Post-PHV for the low jumping activity and no significant change in PVF for the high jumping activity. There were also no significant maturity differences in ALR for both jumping activities in boys, and they tended to have slightly higher force and loading rates in comparison to girls for these activities. Previous studies have demonstrated that pre-pubescent athletes (boys and girls) have higher PVF during jumping activities compared to those who are post-pubertal (36–38). Quatman et al. (24) reported a reduction in PVF during drop landing, but only in boys. They did, however, find a reduction in ALR for both sexes with maturation. The findings of the present study for girls are therefore in agreement with this literature, but there are notable differences for boys. Despite being categorised by biological maturity (Pre- and Post-PHV), which was calculated based on maturity offset, children were recruited into the study based on their chronological age as this was the easiest way for schools to accommodate data collection. As boys reach PHV on average 2 years after girls, the boys who are classified as being post-PHV are therefore likely to be less mature and closer to PHV than girls, who are further past it. This may mean that the advances in growth and maturity that result in improved landing technique (improved lower limb performance, changes in bone geometry, muscle strength and tendon stiffness (22)) are yet to be fully established in boys and therefore have not yet equated to benefits in their jumping performance. In addition, the adolescent growth spurt is also associated with ‘adolescent awkwardness’ which is a temporary disruption of motor coordination that occurs primarily in males during periods of rapid growth (23, 39). The rapid changes in body proportions can disturb proprioceptive abilities (40) and may have contributed to inconsistencies in jumping performance and resulted in the findings observed. This may also be why boys tended to have a higher PVF than girls. Differences in motivation or familiarity with jumping tasks across sex and maturity groups may have also contributed to variation in loading response; however, activities were performed in a pre-randomised order and preceded by practice and familiarisation to reduce potential motivation or order effects. There have been conflicting findings reported in the literature for sex-related differences in jumping activities. For example, both Swartz et al. (38) and Quatman et al. (24) reported a lack of significant sex differences in pre-pubertal, pubertal and post pubertal groups. Erlandson and colleagues (31) also reported no differences in 8–12-year-old boys and girls during a number of different jumping activities. However, in contrast, and in agreement with the trends identified in the present study, others (12, 19, 21) have reported significantly higher GRFs in boys during a range of different jump types. However, these studies are limited by the fact that they have failed to account for biological maturation or have used secondary sex characteristics to compare boys and girls, which does not compare them at a common maturational landmark. Findings must therefore be interpreted with caution and future research is needed to corroborate the findings observed in the present study.

Taken together, findings demonstrate that the effect of maturity on both PVF and ALR differed across activities and between sexes. This therefore indicates that when determining the osteogenic characteristics of PA and developing thresholds to classify impact loading in youth, these may benefit from being tailored according to sex and maturational stage (Pre-/Post-PHV) and should not be a fixed population value, unlike those often applied in adults (e.g., >4BW = high-impact in adults (7, 8);. For example, the ~8 BW·s^−1^ reduction in loading rate between pre- and post-PHV girls during high jumps represents roughly a 25% lower rate of force application, bringing the loading magnitude experienced by post-PHV girls close to that of post-PHV boys performing a *low*-intensity jump. In osteogenic terms, this suggests that maturation alters the mechanical classification of everyday activities and what is high-impact for a younger child may only provide a moderate stimulus in older adolescents, particularly in girls who appear to adopt more force-attenuating movement strategies. Moreover, impacts during activities such as running or jumping rarely occur in isolation; they accumulate over hundreds or thousands of foot strikes or jumps. Therefore, even modest maturational differences in per-impact loading could translate into substantial changes in cumulative skeletal exposure to mechanical strain, amplifying their relevance for bone adaptation across growth. If such maturational changes in osteogenic potential are ignored, there is a risk of underestimating or misinterpreting the skeletal stimulus provided by common movements during adolescence.

Although the maturity- and sex-related differences observed were modest, they are likely to be meaningful when considered in the context of repeated exposure. In school physical education and youth sport, activities such as running and jumping are performed repeatedly within sessions and across weeks or months. Even small per-impact differences in peak force or loading rate (e.g. ~0.2–0.3 BW in PVF or ~5–10 BW·s^−1^ in ALR) may therefore accumulate into substantially different mechanical exposures over time. In sport settings, this has potential relevance for injury risk during growth, particularly during adolescence when rapid changes in body proportions, neuromuscular control and tissue properties may reduce the capacity to tolerate high loading rates (23, 24, 41). The finding that girls showed larger maturity-related reductions in loading during higher-intensity jumping may reflect more force-attenuating strategies, whereas boys tended to maintain or increase loading, which may have implications for how impact-based training or competition loads are progressed across maturation. From a health and intervention perspective, these findings suggest that prescribing identical activities or volumes to children of different sex and maturational status may not result in equivalent mechanical stimuli. Programmes aiming to promote skeletal health during growth may therefore benefit from considering not only the type of activity performed, but also how loading magnitude and rate differ with maturation. In school-based contexts, where activities are often delivered uniformly across age groups, the loading values reported here provide practical reference points for contextualising the mechanical demands imposed by common movements. Together, these findings reinforce that while activity type remains the primary determinant of mechanical loading, sex and maturity influence how that load is experienced, and may help inform interpretation of osteogenic stimulus and musculoskeletal demand during growth in a variety of contexts.

A key strength of this study is the inclusion of a large sample of children and adolescents which enabled sex and maturity-related differences in loading characteristics to be investigated. Characterising the maturity groups based on PHV, a common maturational landmark in boys and girls, is also a strength. Previous studies have either grouped children into maturity groups based on secondary sex characteristics or made comparisons between boys and girls of the same chronological age. There are well documented sex differences in both the tempo and timing of puberty with girls entering and finishing puberty on average two years earlier than boys (42). Comparisons based on chronological age are therefore not appropriate. Further, aligning boys and girls based on secondary sex characteristics assumes that the order and timing of their appearance is identical in both sexes. This is not the case and therefore prohibits effective maturity comparisons (42). Defining maturity relative to PHV provided a biologically meaningful framework for comparison, ensuring that observed differences reflected developmental rather than chronological variation (26, 42). Nevertheless, there are a number of methodological limitations that warrant consideration.

Firstly, the use of portable rather than a fixed laboratory mounted force plate could be considered a limitation. Although lab-based systems are often regarded as the ‘gold standard’ for measuring GRFs, portable platforms have been shown to yield data that is both reliable and precise, and closely matches those obtained from laboratory mounted devices (41, 43–45). In addition, two models of portable force plate were used across data collection waves, operating at different sampling frequencies (200 Hz and 1000 Hz; due to technical specification and device availability). Whilst lower sampling rates can, in theory, reduce the resolution of impact peaks, previous research has demonstrated excellent agreement in peak force estimates collected at 200 Hz compared with higher frequencies (250, 400 and 500 Hz) (46, 47) and minimal differences even at 1200 Hz (48). It is therefore unlikely that differences in sampling frequency or force plate model impacted the force data obtained. Secondly, maturity status was dichotomised into Pre- and Post-PHV groups. This approach does not capture the continuous nature of biological maturation or the variability around the age of PHV. However, the small number of participants at PHV (-0.5 – +0.5) precluded finer categorisation. Despite this limitation, presenting results by distinct maturational stages allowed a clear and interpretable comparison of developmental differences. Larger, longitudinal studies that directly measure, rather than estimate, maturity offset are needed to confirm how loading evolves from childhood through adolescence. Thirdly, the range of included activities was limited to lower-limb, weight-bearing movements performed at a moderate, submaximal intensity, reflecting everyday movement, rather than maximal or sport-specific performance, which is more typically used in laboratory protocols. Although this may have resulted in lower absolute loading magnitudes (23), it provides a more realistic representation of the movements commonly undertaken by children in daily life. In conjunction with the inclusion of an untrained, community-based sample, this enhances the generalisability of findings to the wider paediatric population, rather than the highly trained or athletic cohorts that have often been the focus of previous research. In addition, walking and running were performed over a short indoor runway at fixed cadences, which may differ from free-living outdoor locomotion speeds and patterns and therefore influence absolute loading magnitudes. Finally, although GRFs provide a practical and widely used surrogate of external mechanical loading (28, 29), they do not directly capture internal skeletal strain, which is also influenced by muscle forces, joint kinematics, bone geometry and neuromuscular control, all of which may change with maturation (28).

Study findings demonstrate that, for a given activity, the magnitude and rate of mechanical loading vary according to both sex and maturity. Although modest, these differences suggest that the mechanical stimulus associated with everyday activities may not be entirely equivalent across developmental stages or between boys and girls. Even small differences in peak impact forces and loading rates may influence the skeletal stimulus experienced during growth, particularly when impacts are accumulated repeatedly through habitual activity. The loading values reported here provide empirically derived reference points for a range of everyday movements commonly performed by children and adolescents. These values will assist researchers and practitioners in interpreting the likely mechanical stimulus associated with different activities and in contextualising the osteogenic challenge imposed by physical activity interventions. This is particularly relevant given that most interventions targeting bone health remain poorly characterised in terms of mechanical loading, often relying on non–bone-specific indicators such as heart rate, perceived exertion, or time spent in moderate-to-vigorous physical activity (MVPA), limiting comparability and replicability across studies. At the same time, it is important to recognise that PVF and ALR represent surrogate measures of skeletal loading. Bone adaptation is influenced by a complex interaction between muscular forces, joint moments, strain distribution and the temporal characteristics of loading, many of which also change with growth and maturation. Future research integrating external measures of force with more detailed biomechanical, musculoskeletal or imaging-based approaches (e.g., musculoskeletal modelling) will be important for refining our understanding of how mechanical loading translates to skeletal adaptation across development.

## Data Availability

The raw data supporting the conclusions of this article will be made available by the authors, without undue reservation.
